# 1686. Antimicrobial Activity of Aztreonam-avibactam, Ceftazidime-Avibactam, and Comparator Agents against *Pseudomonas aeruginosa* from Cystic Fibrosis Patients

**DOI:** 10.1093/ofid/ofac492.1316

**Published:** 2022-12-15

**Authors:** Helio S Sader, Leonard R Duncan, Rodrigo E Mendes, Mariana Castanheira

**Affiliations:** JMI Laboratories, North Liberty, Iowa; JMI Laboratories, North Liberty, Iowa; JMI Laboratories, North Liberty, Iowa; JMI Laboratories, North Liberty, Iowa

## Abstract

**Background:**

Aztreonam-avibactam (ATM-AVI) is under clinical development for the treatment of serious infections caused by Gram-negative bacteria, including MBL producers. Four other β-lactamase inhibitor combinations (BL/BLI) have been recently approved by the US FDA: ceftazidime-avibactam (CAZ-AVI), ceftolozane-tazobactam (C-T), meropenem-vaborbactam (MEM-VAB), and imipenem-relebactam (IMI-REL). We evaluated the *in vitro* activity of these 5 BL/BLIs and comparators against *P. aeruginosa* (PSA) causing cystic fibrosis (CF) pulmonary exacerbation.

**Figure:**
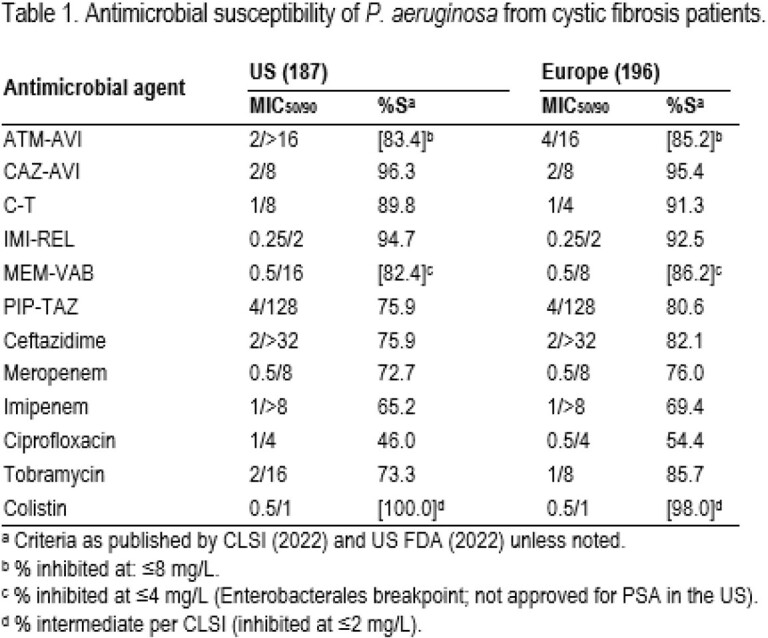

**Figure:**
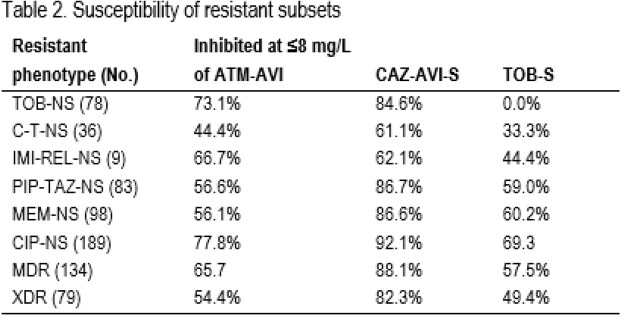

**Methods:**

383 PSA isolates (1/patient) were recovered from 35 medical centers in the US (*n*=187) and 12 European countries (EU; *n*=196) in 2018-2021 and susceptibility tested by CLSI broth microdilution method.

**Results:**

ATM-AVI inhibited 83.4%/85.2% of isolates from US/EU at ≤8 mg/L. CAZ-AVI (MIC_50/90_, 2/8 mg/L; 96.3%/95.4% susceptible [S] in US/EU) was the most active agent, followed by IMI-REL (94.7%/92.5%S in US/EU) and C-T (89.8%/91.3%S in US/EU). MEM-VAB (not approved for PSA in the US) and MEM showed similar activity. Tobramycin (TOB)-S rates were 73.3%/85.7% in US/EU (Table 1). Among TOB-non-S (NS) isolates, 84.6% were CAZ-AVI-S and 73.1% were inhibited at ATM-AVI MIC of ≤8 mg/L. CAZ-AVI retained good activity against C-T-NS (61.1%S), IMI-REL-NS (62.1%S), piperacillin-tazobactam (PIP-TAZ)-NS (86.7%S), meropenem (MEM)-NS (86.6%S), and ciprofloxacin (CIP)-NS (92.1%S) isolates (Table 2). Multidrug-resistant (MDR) and extensively drug-resistant (XDR) phenotypes were observed among 40.1%/30.1% and 24.6%/16.8% of isolates from US/EU, respectively. Among MDR isolates, 90.7%/84.7% were CAZ-AVI-S, 65.3%/66.1% were inhibited at ≤8 mg/L of ATM-AVI, and 78.7%/76.3% were TOB-S in US/EU. CAZ-AVI and TOB retained activity against 87.0%/75.8% and 39.1%/63.6% of XDR isolates in US/EU, respectively.

**Conclusion:**

ATM-AVI and CAZ-AVI exhibited potent activity against PSA isolated from CF patients in US and EU and retained good activity against isolates resistant to other antimicrobials, including MDR and XDR organisms; both compounds showed greater activity than TOB. ATM-AVI and CAZ-AVI may represent a valuable option to treat CF patients with pulmonary exacerbations due to PSA infection.

**Disclosures:**

**Helio S. Sader, MD, PhD**, AbbVie: Grant/Research Support|Cidara: Grant/Research Support|Melinta: Grant/Research Support|Nabriva Therapeutics: Grant/Research Support|Pfizer: Grant/Research Support **Leonard R. Duncan, PhD**, AbbVie: Grant/Research Support **Rodrigo E. Mendes, PhD**, AbbVie: Grant/Research Support|Cidara: Grant/Research Support|GSK: Grant/Research Support|Melinta: Grant/Research Support|Nabriva Therapeutics: Grant/Research Support|Office for Assistant Secretary of Defense for Health Affairs: Grant/Research Support|Pfizer: Grant/Research Support|Shionogi: Grant/Research Support|Spero Therapeutics: Grant/Research Support **Mariana Castanheira, PhD**, AbbVie: Grant/Research Support|Cidara: Grant/Research Support|GSK: Grant/Research Support|Melinta: Grant/Research Support|Pfizer: Grant/Research Support|Shionogi: Grant/Research Support.

